# Comparative analysis of emergency department admissions: A multi-center study on patient characteristics and mortality before and during the early phase of pandemic in Turkey

**DOI:** 10.1097/MD.0000000000044438

**Published:** 2025-09-12

**Authors:** Emre Şanci, Başak Bayram, Ahmet Naci Emecen, Asim Enes Özbek, Emrah Çelik, Onursal Varlikli, Şenol Ergüney, Alper Akin Gözübüyük, Hüseyin Cahit Halhalli

**Affiliations:** a Department of Emergency Medicine, Bakircay University Faculty of Medicine, Cigli Training and Research Hospital, İzmir, Turkey; b IBB Izmir Esrefpasa Hospital, İzmir, Turkey; c Department of Public Health, Dokuz Eylul University Faculty of Medicine, Epidemiology Subsection, İzmir, Turkey; d Department of Emergency Medicine, Kocaeli City Hospital, Kocaeli, Turkey; e Department of Emergency Medicine, Emergency Medicine Specialist, Kocaeli City Hospital, Kocaeli, Turkey; f Department of Pediatric Surgery, Kocaeli University, Faculty of Medicine, Kocaeli, Turkey; g Department of Urology, Buyuk Anadolu Hospital, Kocaeli, Turkey; h Department of Infectious Diseases, Kocaeli Derince Training and Research Hospital, Kocaeli, Turkey.

**Keywords:** admissions, COVID-19, emergency departments, mortality, pandemic

## Abstract

The effect of the global healthcare events makes its initial impact to the emergency departments. As the vanguards of healthcare system emergency departments had handled the surge while undergoing physical changes to manage ongoing crisis. Therefore, for the preparation of further global events, admission rates and emergency department utilization of the pandemic requires further investigation. The aim of this study was to analyze the admissions in a multi-center scale and compare ICD codes and crude mortality rates during the early COVID-19 pandemic period and compare this data to the previous year of the outbreak. This was a multi-center study of 10 hospitals; including 2 tertiary and 8 secondary healthcare institutions. This study comparatively analyzed ED admissions between during pandemic and pre-pandemic periods. A total of 8,38,502 admissions were included in the study (5,72,443 vs 2,66,059; pre pandemic period vs during pandemic period, respectively). There was a significant difference between age, gender, ED waiting time, triage color, and hospitalization rates (*P* < .001) between 2 periods. Comparison of admission international statistical classification of diseases and related health problems 10th revision codes were significantly different between pre-pandemic and pandemic periods for trauma, cardiovascular, neurology, and respiratory codes (*P* < .001, *P* < .001, *P* < .001, *P* = .024, respectively). The findings of this study suggest that a pandemic affects many mechanisms in the emergency healthcare systems. This influence might have affected the ED admission rates, hospital admission rates, and mortality rates.

## 1. Introduction

Emergency departments (EDs) act as the vanguards of the healthcare system and its role is heightened during crisis periods. For public healthcare emergencies and pandemics to be managed properly, past crisis’s data should be evaluated rigorously for strengthening preparedness and planning. Recent pandemic had a major impact on emergency medicine systems as well as having social effects worldwide, facility designs and disrupted the operations of healthcare facilities left a lesson to be remembered^[1,2]^ (SDC 1, SDC 2, Supplemental Digital Content, https://links.lww.com/MD/P925). Unprecedented levels of critically ill patients have caused management challenges, especially in the EDs.

Despite the surge in critically ill patients with COVID-19, a substantial fall in the presentation of patients with non-COVID has been reported in many countries.^[3–6]^ Although most of these presentations consist of nonurgent medical conditions, some of these have been cases that immediately seek care for serious conditions including ST elevation myocardial infarction, stroke, syncope, chronic respiratory diseases, heart failure, gastrointestinal hemorrhage, trauma, and other severe diseases.^[7,8]^ The meta-analysis reported that the number of stroke alerts, reperfusions, and mechanical thrombectomies reduced during the pandemic.^[9]^ Additionally, a decrease in the number of patients with acute coronary syndrome and a delay in their presentations have been reported.^[10,11]^ This reduction might be attributable to fear of exposure to COVID-19 in healthcare settings as well as the implementation of extensive protective public health and social measures for COVID-19 prevention and control.^[12,13]^ EDs have an integral role in managing these acutely ill patients therefore, delayed care for these acute conditions and exacerbations of chronic diseases might lead patients to significant risk for preventable morbidity and mortality.

In Turkey, the first case of COVID -19 was identified on March 11, 2020. Ever since the number of confirmed infections increased exponentially during pandemic period, afterwards, strict public health, and social measures have been implemented to limit viral transmission.

Most importantly, precautions for healthcare facilities have been taken. EDs have been restructured urgently in many aspects. Maintenance of a safe environment for health care staff has been provided, appropriate personal protective equipment has been provided for health care workers, pre-triage systems have been established outside most of the EDs, potentially contaminated and clean areas have been divided and reshaped.

In Turkey, the ED visit rate is 140 visits per 100 people whereas, in the US the rate is 40 visits per 100 people.^[14,15]^ Most of these presentations consist of nonurgent medical conditions.^[16]^ According to the study conducted in a single urban hospital in Turkey, ED visits decreased by 48% of the previous year during the pandemic.^[5]^ ED physicians have proficiency in management of the patients with acute illnesses therefore, it is essential to investigate the effect of the drop in the number of ED visits during the pandemic period.

Kocaeli is the second most densely populated city in Turkey. By location and population Kocaeli represents geographically diverse areas in Turkey. Immigrants from many rural and urban areas of the country and other countries as Syria compose the population of the city. There have been nineteen hospitals in Kocaeli, ten of which are state hospitals that manage 85% of the ED visits of the city. Thus, investigation of the variation in number of ED visits and mortality rates in this cosmopolitan city will contribute to the management of the patients during the pandemic period, estimating the mortality risk and predicting the progression of the pandemic.

The aim of the study was to examine the impact of the current COVID-19 outbreak on the number of ED visits and also in-hospital mortality rates in ten state hospitals when compared to the same period in the previous year. To our knowledge, this is the most comprehensive study that examine the impact of the pandemic on ED visits and hospital mortality rates in Turkey.

## 2. Methodology

### 2.1. Study design and setting

ED admissions between March 11 and May 20 for 2019 and same timeline for 2020 were acquired and analyzed from 10 public hospitals (2 tertiary and 8 secondary healthcare centers). Data of ED admissions of March-April-May of 2019 have been evaluated as “pre pandemic” period while data of March-April-May of 2020 have been evaluated as “during pandemic” period. “During pandemic” period timeline has been selected to reflect the first peak of the pandemic in Turkey (Figure S1, Supplemental Digital Content, https://links.lww.com/MD/P924).^[17]^ The data has been acquired from Provincial Health Directorate of Kocaeli with approvals in August of 2020 without any information that could identify individual participants.

Administrative data records of ED admission including international statistical classification of diseases and related health problems 10th revision (ICD-10) codes, admission to hospital wards, ED waiting times, triage color codes, and mortality rates have been evaluated.

ED waiting times have been defined as the time between record of admission time and input of ICD-10 code. In-hospital mortality has been defined as death of any cause occurred in these hospitals as a crude mortality rate.

This study has been approved by The Institutional Ethics Committee of Kocaeli Derince Training and Research Hospital (2020-69). This study was conducted in accordance with the Declaration of Helsinki.

### 2.2. Outcome measures

Data have been divided and compared between pre-pandemic and during pandemic periods. The primary outcome was to evaluate the ED admissions during and prior to the pandemic period. The secondary outcome measure is to compare hospital admissions and mortality rates.

### 2.3. Statistical analysis

Continuous variables were expressed as mean ± SD and Compared with Mann–Whitney *U* test. Categorical variables will be presented as percentages and evaluated for their conformity to the normal distribution and analyzed using the Wilcoxon test. All statistical analyzes were performed using the program “R” and a *P* value <.05 was considered statistically significant.

## 3. Results

A total of 8,38,502 admissions were included in the study (5,72,443 vs 2,66,059; pre pandemic period vs during pandemic period, respectively). In the pandemic period ED admission had lower ED waiting times, fewer patient triaged in each triage code and less hospitalization rates. Summary of age, sex, ED waiting times, and triage color codes have been visualized and compared in Table 1.

**Table 1:** The patients’ admission characteristics between periods.

	Pre-pandemic	During pandemic	n	*P* value
Sex				
Male	2,82,898 (49.4%)	1,49,434 (56.2%)	8,38,502	<.001
Female	2,89,545 (50.6%)	1,16,625 (43.8%)		
Age	32.0 (21.6)	37.5 (20.0)	8,38,502	<.001
ED wait time (min)	15.8 (66.5)	13.4 (83.1)	8,37,451	<.001
Triage color				
Red	13,352 (2.49%)	16,550 (6.51%)	791,503	<.001
Yellow	3,47,587 (64.7%)	1,70,280 (67.0%)		
Green	1,76,244 (32.8%)	67,368 (26.5%)		
Black	64 (0.01%)	58 (0.02%)		
Hospital admission				
No	5,63,581 (98.5%)	2,56,547 (96.4%)	838,502	<.001
Yes	8862 (1.55%)	9512 (3.58%)		
Institiution grade				
Secondary	3,58,669 (62.7%)	1,88,611 (70.9%)	838,502	.001
Tertiary	2,13,774 (37.3%)	77,448 (29.1%)		

Admission ICD-10 codes compared between periods; trauma, cardiovascular, neurology, and respiratory admission were significantly lower in early pandemic period (*P* < .001, *P* < .001, *P* < .001, *P* = .024, respectively). ICD-10 codes for ED admissions have been summarized in Table 2.

**Table 2:** ED Admissions’ ICD-10 codes.

	Pre-pandemic	During pandemic	*P* value
Trauma (ICD S, T, W)			
No	5,44,230 (95.1%)	2,56,123 (96.3%)	<.001
Yes	28,213 (4.93%)	9936 (3.73%)	
Cardiovascular (ICD I)			
No	5,70,655 (99.7%)	2,64,916 (99.6%)	<.001
Yes	1788 (0.31%)	1143 (0.43%)	
Neurology (ICD G)			
No	5,70,459 (99.7%)	2,64,966 (99.6%)	<.001
Yes	1984 (0.35%)	1093 (0.41%)	
Respiratory (ICD J)			
No	5,53,327 (96.7%)	2,56,920 (96.6%)	.024
Yes	19,116 (3.34%)	9139 (3.43%)	

In-hospital mortalities between periods for gender and age have been evaluated and summarized in Table 3. ED admissions were significantly lower during pandemic periods (Figure S2, Supplemental Digital Content, https://links.lww.com/MD/P924).

**Table 3:** In-hospital mortalities (crude mortality rate) between periods.

	Pre-pandemic (n = 495)	During pandemic (n = 729)	*P* value
Sex			
Male	239 (48.3%)	435 (59.7%)	<.001
Female	256 (51.7%)	294 (40.3%)	
Age	72.3 (16.3%)	70.5 (17.4)	.056

## 4. Discussion

The results of the study showed that the number of admissions to EDs decreased exceptionally during the pandemic period. In contrast, during this period both the number of hospital admissions and admission rates increased. Additionally, the number of deaths augmented tremendously during the pandemic period.

Present study aimed to reflect the first peak on pandemic and its effects on ED admissions since in early phases of the pandemic, medical community had no practical easy-to-use diagnostic tests and limited algorithmic approach in the diagnosis and treatment of these patients. Additionally, the general population had the fear of contacting with disease and strict curfews were in place to implement social distancing to reduce transmission. This results were similar to other similar studies exploring ED admission.^[12,13]^

In this study, the effect of the pandemic on the number of ED admission was evaluated and compared with the previous year’s same period. By this way, the effect of seasonal changes on ED visits was eliminated. Before the pandemic, the number of ED admissions was between 7000 and 9000 in a week whereas, during the pandemic after the first confirmed case of coronavirus the number of ED admissions plunged. Especially during lockdown periods, the city-wide number of ED visits in a week dropped by under 2000 admissions in a week (Figure S1, SDC 2, Supplemental Digital Content, https://links.lww.com/MD/P924; https://links.lww.com/MD/P925).

The decline in the number of ED admissions might be attributable to a few reasons. Firstly, patients with nonemergency conditions might have avoided seeking care due to the restrictions during the lockdown and fear of getting infected.^[18]^ Secondly, even patients with life-threatening conditions might have been reluctant to seek medical help.^[19]^ Thirdly, other alternatives such as telemedicine might have displaced emergency admissions.^[20]^ Lastly, the precautions to prevent the spread of COVID-19 might have reduced the prevalence of other infectious diseases.^[21]^ According to the results of this study, the decline in the number of ED admissions during the pandemic occurred especially in patients who were given green and yellow triage codes. The number of applications has dropped to one-third and half of the pre-pandemic level, respectively. Whereas both the number and rate of patients with red triage codes increased during the same period. This rise could be ascribed to the pandemic. Furthermore, the fall in the number of yellow triage codes might be the indicator of disruption of health care services due to the pandemic.

As stated by the results of this study the number of ED admissions to tertiary care centers dropped off more than the secondary care centers. During the pandemic, some of the health facilities in the city were designed to take care of only patients infected with COVID-19. Therefore, this precaution might have affected the admission rates of some of the tertiary care centers. Additionally, owing to the fact that tertiary care centers are hospitals where more complicated patients are followed and treated, these facilities might have been perceived as more dangerous for the spread of COVID-19.

In spite of the fact that the number of ED admissions plunged during the pandemic, contrary to previous literature, the number of hospital admissions increased during the pandemic period.^[19,22]^ In addition, the hospital admission rate increased twice from the previous year. This might be attributable to the difference in the management of infected patients during the pandemic among the countries. In Turkey, especially during the beginning of the pandemic, regardless of their health conditions, most of the confirmed COVID-19 cases were followed up in hospitals. This approach might have caused the high admission numbers and rates during the pandemic. However, in accordance with the previous studies, the number of hospital admissions due to cardiovascular, neurologic, and respiratory illnesses declined remarkably during the pandemic.^[23–25]^ This shows the justification of the interpretation of this issue.

In this study, the early stages of the pandemic were compared with the previous year. The previous studies reported that especially during the early stages of the pandemic number of traumatic injuries decreased.^[26,27]^ In accordance with the previous studies, in this study, the volume of patients who were admitted to the ED due to traumatic injuries dropped to one-third of the previous year. Stay-at-home orders and remote working could have contributed to this decline.

Although this study compared the pandemic period with the previous year’s data, these 2 periods of time may not be suitable for comparison in the lockdown conditions. The pandemic was a period in which people worked remotely, fewer traffic accidents occurred, and lower risk of infectious diseases which all may contribute to the low mortality rates. Therefore, expected mortality rates were lower than the previous year. However, according to the results of this study, the mortality rates boosted rapidly during the pandemic period. Additionally, the increase in number of deaths among male gender was 5 times higher than females. The results of this study were compatible with the previous studies.^[28,29]^ Excess deaths could be attributed to the delayed effects of COVID-19.^[28]^ Furthermore, the decreased number of admissions due to cardiovascular, neurologic, and respiratory illnesses might have contributed to this augmentation.

## 5. Limitations

The study had several limitations. Firstly, the retrospective nature of the study might have affected the results of the study. Secondly, the comorbidities are associated with worse outcomes for patients confirmed with coronavirus.^[30]^ The lack of data about the comorbidities of the patients, the results of the study might have been affected. However, the large sample size minimizes the margin of error arising from this limitation. Thirdly, although this study was conducted in ten public hospitals in one of the biggest cities in Turkey, the results of the study can’t be generalized worldwide considering every nation had its own different approaches to the pandemic. Lastly, this study explored the early phase of the pandemic. The pandemic had different attributes throughout its phases and explorations other than first peak of the disease might yield different results. Furthermore, inclusion of admission patterns of the following years of pandemic might have yielded different perspectives.

## 6. Conclusion

In conclusion, number of ED visits during the first peak of pandemic were significantly lower to the previous year and crude mortality rates were higher. This can be attributed to the limited knowledge about managing the pandemic during early phases. Further research exploring aspects of admissions and utilization of EDs during different phases of pandemic would bring insight for administrative efforts in future pandemics.

Supplemental digital content “SDC3” is available for this article (https://links.lww.com/MD/P926).

## Author contributions

**Conceptualization:** Emre Şanci, Başak Bayram.

**Data curation:** Emrah Çelik, Alper Akin Gözübüyük, Hüseyin Cahit Halhalli.

**Formal analysis:** Ahmet Naci Emecen.

**Investigation:** Ahmet Naci Emecen, Emrah Çelik, Alper Akin Gözübüyük.

**Methodology:** Alper Akin Gözübüyük, Hüseyin Cahit Halhalli.

**Project administration:** Onursal Varlikli, Şenol Ergüney.

**Software:** Ahmet Naci Emecen.

**Supervision:** Emre Şanci, Onursal Varlikli, Şenol Ergüney, Hüseyin Cahit Halhalli.

**Visualization:** Emre Şanci.

**Writing – original draft:** Emre Şanci, Asim Enes Özbek.

**Writing – review & editing:** Başak Bayram, Asim Enes Özbek.

## Supplementary Material


