# Supplementary material for: Comparative analysis of emergency department admissions: A multi-center study on patient characteristics and mortality before and during the early phase of pandemic in Turkey
Source: Medicine (Baltimore). 2025 Sep 12;104(37):e44438. doi: 10.1097/MD.0000000000044438 (PMC12440398; doi:10.1097/MD.0000000000044438)
Supplement: Supplementary file 1 [file medi-104-e44438-s001.pdf]

NATIONAL STRATEGIC PLAN FOR EMERGENCY DEPARTMENT MANAGEMENT OF OUTBREAKS OF COVID-19

Purpose

This plan informs health care personnel, public health, and government officials at all levels of the necessary capabilities that must be present for successful emergency department management of an outbreak of COVID-19. It also enumerates the necessary actions that must be taken to attain these capabilities. The performance of the necessary actions will fall to parties including professional associations, government entities at the federal, state and local levels, public health officials and departments at the federal, state and local levels, and hospitals – administrators, medical staff, nursing and support services. Those entities should undertake the necessary operational planning to assure their performance prior to, during and following an outbreak of COVID-19. The plan serves as a guide for emergency departments to address the interdependencies that are necessary for successful management of such an incident. The goal of this guidance is to protect the health care infrastructure and ensure the delivery of emergency medical treatment during a large-scale epidemic or pandemic.

Background

COVID-19 is an emerging coronavirus initially discovered in Wuhan, China in December, 2019. The origin of this virus is currently unclear, although it may be a zoonotic spillover virus from the bat order, Chiroptera, possible passing through one or more other species before infecting humans. The overall picture of this infectious disease is changing rapidly with much still unknown about its modalities of spread, incubation and infectious periods and other important aspects of the virus. However, at this point in time, some aspects of this virus are worrisome: 1) it appears to be very contagious and able to spread rapidly by respiratory droplet spread, similar to influenza; 2) there is concern that asymptomatic patients harboring the virus may still be able to spread the infection to others; and 3) the current case-fatality rate in symptomatic patients appears high, however when mildly symptomatic or asymptomatic patients are taken into account, the CFR is probably much lower overall. Currently, as of this writing (March 1, 2020), COVID-19 has 88,587 confirmed cases worldwide (most in China) with 3,038 deaths with infected patients in more than 66 countries/regions. The World Health Organization has classified this disease as globally very high risk,.

Risk Awareness

An important community mitigation strategy is keeping people who are ill from entering the workplace and school. Encouraging social distancing and frequent hand washing policies are important parts of the strategy. The combination of the effects of the disease and employing these measures could affect the business practices of all the critical infrastructure operators, possibly impeding their ability to maintain normal operations.

At some critical point the operations of hospitals may be affected, not only by the absence of hospital workers, but by slowdowns in transportation of critical supplies and support services, the effect of these factors being the inability to maintain normal operations, even for normal patient volumes. Each year, normal influenza season can stress an emergency department's ability to maintain normal operations because of the increase in both outpatient volume and admissions. It is therefore prudent to prepare for a worse than normal influenza season. While the precise effects of COVID-19 on emergency departments' ability to function cannot be predicted with complete confidence at this time, contingency plans should be made for a challenging scenario.

Vulnerability

Population. It is unclear at this time as to which populations may be more vulnerable to COVID-19, although initial data suggests that elderly individuals (especially males) and those with underlying medical problems may be more at risk for a bad outcome from this infection.

Critical Infrastructure

The ability of some critical infrastructures components to continue normal operations depends largely on the attendance of its workforce. Others may have plans and procedures in place for tele-work or social distancing. The nation's just-in-time supply chain could experience delays in supplying goods and services, and businesses who cannot survive operational interruptions may expand inventories of supplies. Hospitals are no exception to this. Pharmacies may not be able to get additional supplies from distributors or sources higher in the chain, domestic and foreign.

Supplies of personal protective equipment (PPE) and other supplies may require stockpiling, and food services could encounter supply problems due to impaired production and delivery. The workforce may be depleted due to infection, fears of coming to work in an infectious environment, or the need to care for children out of school or other family members. Medical care is highly labor intensive; thus, service delivery is expected to be exquisitely sensitive to the ratio of service demand to workforce supply.

Consequence

The consequences to society will vary with the severity of the illness (ease of transmission and virulence), the degree to which the population is prepared and resilient (vaccinated- if and when an effective vaccine becomes available, compliant with community mitigation strategies, and educated about the threat), and whether business and industry can sustain productivity during a pandemic. A COVID-19 outbreak with the severity of the 1918 influenza pandemic obviously could have far-reaching consequences. Lesser-severity outbreaks could result in temporary disruptions in the flow of goods and services and would likely cause further stresses on an already over-burdened health care system. Certain medical services could be delayed or unavailable, potentially causing secondary morbidity and mortality to those unaffected COVID-19. A milder COVID-19 outbreak than feared will result in charges of over-reaction and fear mongering, which could have detrimental effects on future compliance with public health measures, future immunization initiatives and compliance with a response to public health emergencies.

There are consequences to planning, including financial investment and diversion of human resources, and consequences to the lack of planning should the threat become reality. In either case, a prudent assessment of risk is vital and prudent planning in response to that risk is necessary to mitigate possible consequences.

Response to the Threat

The American College of Emergency Physicians (ACEP) supports comprehensive response planning by its members and their institutions. ACEP will work with its corresponding professional associations (e.g. nurses, prehospital professional, EMS medical directors, hospitals, public health associations) to implement the actions and satisfy the requirements of the planning process. ACEP will seek support as necessary on behalf of its members from federal government entities responsible for surge medical response capabilities.

ACEP state chapters should seek the involvement of their state public health directors. Members should involve local emergency managers, political leaders and local health directors. The national strategic plan planning process and the capabilities and actions herein should prompt a local process that defines how to operationalize these capabilities and to articulate the necessary requirements to perform them. Those requirements should result in the targeting of resources and the identification of gaps that may exist in funding. Those unfunded requirements must be communicated to local emergency managers and political leaders to guide resource allocations and possible supplementary grant funding from federal entities.

Emergency physicians and their professional colleagues in nursing and prehospital care will be on the front line of any emergency requiring surge medical response capability. They will be the ones to answer the challenge of waves of patients presenting for care. They will also be the beneficiaries of good planning or the victims of the lack of it. ACEP will exercise all of its influence to ensure that patients can be cared for and that its members are sufficiently resourced, trained, equipped and exercised to fulfill their critical mission. Triggers for various parts of a response plan are part of the planning process, and the authorities to take action must be clear. Likely, as cases are recognized and increasing strain is put on acute care facilities, the health care professionals and administrators in those facilities will be the first to know there is a crisis.

Communications with local and state public health directors for situational awareness are of paramount importance. Clear communications protocols must be created and exercised on a regular basis prior to an event. Agreements on stepwise implementation of the relevant parts of the plan must be in place to avoid disruptions in services during the ramp-up phase and to allow for the necessary provision of resources. Just as the parts of the plan will be implemented with the situational awareness of the local leaders and public health officials, so will the "standing down" of the pandemic plan. Agreements must be in place as to the triggers for the authorities to return to the prior steady state. This decision must be made with situational awareness of the epidemiology of the disease in the community and cannot be done, institutionally, in a vacuum.

Depending on the severity of the outbreak and its virulence, institutions may face a depleted workforce. In addition to those lost to illness, there may be those with concerns about being in an environment of higher risk from exposure to infectious disease. There may be personnel who have temporary reasons for not returning to the workplace, such as pregnancy or needing to care for family who remain ill. ACEP will work with its counterpart professional associations and the federal government to dispense actionable information to its members so that they remain aware of current information on the changing situation and can make decisions accordingly.

Management Strategy

ACEP will use all means necessary to (1) ensure that our patients receive the best care possible, and (2) ensure that its members are able to fulfill their professional responsibilities. The National Strategic Plan for Emergency Department Management of Outbreaks of COVID-19

follows the federal template for management of biological threats, as outlined in Homeland Security Presidential Directive #10. The pillars of that strategy are: Threat Awareness, Protection and Prevention, Surveillance, Detection, Response and Recovery.

This National Strategic Plan for ED Management of COVID-19 outbreaks adapts this template to form its own pillars of prescribed capabilities:

- Situational awareness
- Protection of the emergency department infrastructure and personnel
- Prevention of disruptions in service delivery
- Organized, timely surge medical response
- Recovery to the previous steady state

Capabilities for Emergency Department Response to a Severe COVID-19 Outbreak

1. Trained Emergency Manager or Chief Preparedness Officer designated as lead for COVID-19 preparedness and response, fully integrated with community emergency preparedness, public health and resource managers
2. Seamless connectivity with local/state governmental emergency management, public health, other hospital Chief Preparedness Officers, and any other support organizations
3. Emergency operations plan for COVID-19
4. Surge staffing plan for the entire institution
5. Hospital Incident Command System and National Incident Management System training, knowledge and compliance
6. Functional Hospital Command Center
7. Training and exercise program for all involved personnel
8. Appropriate PPE for health care staff
9. Capability to screen and test staff for illness
10. Enhanced facility security and crowd management
11. Administrative and legal support
12. Antiviral prophylaxis and vaccine availability for Staff when available and recommended by the CDC
13. Interoperable communications system (fire, law enforcement, EMS, emergency management, receiving hospitals, local/regional public health, local EOC)
14. Maintaining EMS operations during COVID-19 outbreak
15. Laboratory testing protocols
16. Alternate locations and staffing for triage and medical screening exams
17. Off-Site vaccine administration when available and indicated
18. Health information call centers
19. Configuration of ED waiting rooms for distancing to the degree possible
20. Protocols for those visiting patients with fever and respiratory symptoms
21. Environmental decontamination capability
22. Off-site mass screening capability
23. Adequate inpatient surge capacity including the establishment of alternate care facilities
24. Trained and credentialed volunteers
25. Awareness of strategic national stockpile (SNS) surge supplies and equipment and capability to receive those supplies
26. Accurate and coordinated public information dissemination including when to seek care for illness
27. Augmented post-mortem and mortuary services

Critical Actions

Planners for surge medical response should consider the actions in Annex 1 to achieve the necessary capabilities. The method for performance of the actions and the measures of performance will be determined locally. The resulting requirements list (including those currently present and funded, present and not funded, and not present) will guide resource allocations and requests for additional resources.

This plan defines roles and responsibilities that belong to national emergency medicine organizations, such as ACEP, and to each emergency department. It also suggests roles and responsibilities for those with whom interdependencies exist for surge medical response. It is critical, working together locally, to define those roles, articulate the requirements – funded and unfunded, and to quantify and identify the resources required to accomplish them.

1. Trained Emergency Manager or Chief Preparedness Officer designated as lead for COVID-19 preparedness and response, fully integrated with community emergency preparedness, public health and resource managers.

	EM National	Federal Gov.	State & Local Gov	State & Local PH	ED	Hosp.
**Action:**						
a. Designate an in-house position, or new hire (NIMS certified, HICS trained); National Incident Management System (NIMS) Hospital Incident Command System (HICS)						●
b. Establish authority to carry out responsibilities;						●
c. Execute/implement ASPR Influenza Surge Preparedness Assessment as appropriate https://www.acep.org/globalassets/uploads/uploaded-files/acep/clinical-and-practice-management/resources/publichealth/h1n1/aspr-influenzasurgepreparednessassesment.pdf					●	●
d. For institutions that are part of a hospital system, establish/strengthen connections amongst the different emergency managers and hospital leaders						●
e. Review National Guidance for Healthcare System Preparedness https://www.phe.gov/preparedness/planning/hpp/reports/documents/capabilities.pdf					●	●
f. Review/implement AHRQ Mass Medical Care with Scarce Resources: A Community Planning Guide as appropriate https://web.mhanet.com/AHRQ_mass_care_guide11.06.pdf					●	●
g. Maintain awareness of status or threat of COVID-19 in US, state, and region as reported by CDC and state, keeping hospital in posture of preparedness prior to initiation of any emergency operations<br>Review Crisis Standards of Care and its implications for the institution https://asprtracie.hhs.gov/technical-resources/63/crisis-standards-of-care<br>Allocating Scarce Resources in Disasters: Emergency Department Principles Hick, John L. et al. *Annals of Emergency Medicine*, Volume 59, Issue 3, 177 - 187<br>WHO https://www.who.int/docs/default-source/coronaviruse/situation-reports/20200228-sitrep-39-covid-19.pdf?sfvrsn=aa1b80a7_2<br>CDC https://www.cdc.gov/coronavirus/2019-ncov/locations-confirmed-cases.html#map	●	●	●	●	●	●

2. Seamless connectivity with local/state governmental emergency management, public health, other hospital Chief Preparedness Officers, and any other support organizations

	EM National	Federal Gov.	State & Local Gov	State & Local PH	ED	Hosp.
**Action:**						
a. Agree on modalities, nodes, thresholds and frequency for multi-directional information flow/communication during emergency operations such as EOC dash boards.			●	●	●	●
b. Agree on and establish common language and formats for data sharing during emergency operations		●	●	●	●	●
c. Agree on types, degrees (and limitations) of participation in regionalized response to the public health emergency			●	●	●	●
d. Confirm that emergency management/public health authorities have coordinated with clinics, private medical practices, extended care facilities, local medical societies, health care coalitions re: response plan			●	●		
e. Subscribe to the CDC and your state HAN network or other state department of health communication modality	●			●	●	●
f. Confirm connectivity of key subject matter experts (SMEs) and authorities within hospital with their outside counterparts (e.g. hospital laboratories with state labs and Laboratory Response Network facilities) and reporting relationships in-house for common operating picture						●

3. Emergency operations plan for COVID-19

	EM National	Federal Gov.	State & Local Gov	State & Local PH	ED	Hosp.
**Action:**						
a. Appoint a hospital COVID-19 planning group						●
b. Review the hospital's emergency operation plan (EOP) for applicability to COVID-19				●	●	●
c. Utilize Web resources to identify and disseminate model EOPs for COVID-19	●	●	●	●	●	●
d. Review the best plans and adapt to your institution for e.g.<br>**Hospital Disaster Preparedness Self-Assessment Tool**<br>https://www.acep.org/globalassets/uploads/uploaded-files/acep/clinical-and-practice-management/ems-and-disaster-preparedness/hospital-disaster-preparedness-self-assessment-tool.doc					●	●
e. Assess your COVID-19 EOP using a table-top exercise with hospital staff including physicians, administrators and logistics experts present to ensure that the plan is workable and will maintain operations under anticipated circumstances.					●	●

5. HICS and NIMS knowledge and compliance

	EM National	Federal Gov.	State & Local Gov	State & Local PH	ED	Hosp.
**Action:**						
a. Provide HICS/NIMS training for all hospital staff appropriate to their assigned positions<br>https://www.calhospitalprepare.org/hics<br>https://training.fema.gov/nims/						●
b. Ensure that those managing an incident have appropriate levels of NIMS certification:<br>• Incident management leadership - NIMS IS 700<br>• Individuals responsible for the emergency plan - NIMS IS 800<br>• Personnel who have a direct role in middle management and/or emergency response - IS 100. and 200.<br>https://training.fema.gov/nims/			●	●		●

6. Functional Hospital Command Center

	EM National	Federal Gov.	State & Local Gov	State & Local PH	ED	Hosp.
**Action:**						
a. Review and customize hospital command centers (HCC) functions in HICS for your institution<br>https://emsa.ca.gov/disaster-medical-services-division-hospital-incident-command-system-resources/						●
b. Establish authority and criteria to activate the HCC						●

7. Training and exercise program for all involved personnel

Action:	EM National	Federal Gov.	State & Local Gov	State & Local PH	ED	Hosp.
a. Create and execute a training program based upon your emergency operations plan			●			●
b. Develop a template for a COVID-19 exercise program	●	●				
c. Execute an exercise to test training and plan validity			●	●		●
d. Use results of the exercise to further improve the emergency operation plan, and then re-exercise						●
e. Design and implement special training for public affairs in existence of plan to care for all who need it during COVID-19 emergency, changes to normal daily operations that could occur during pandemic, where and how they will get information						●
f. Discuss with hospital public affairs and public health authorities when and how to inform the public as to when and how to access information and care during an outbreak.				●		●

8. Appropriate PPE for health care staff

Action:	EM National	Federal Gov.	State & Local Gov	State & Local PH	ED	Hosp.
a. Consult national guidance for recommended PPE use for patients with confirmed or under investigation for COVOD-19 in healthcare settings https://www.cdc.gov/coronavirus/2019-ncov/infection-control/control-recommendations.html?CDC_AA_refVal=https%3A%2F%2Fwww.cdc.gov%2Fcoronavirus%2F2019-ncov%2Fhcp%2Finfection-control.html					●	●
b. When indicated by the CDC, estimate PPE needs for multiple waves of COVID-19.					●	●
c. Stockpile appropriate quantities of PPE prior to outbreak. Developing a range of respirator conservation strategies, including strategies to make supplies last longer (such as using alternative products like reusable respirators) and extending the use of disposable respirators.<br>Strategies for Optimizing the Supply of N95 Respirators https://www.cdc.gov/coronavirus/2019-ncov/hcp/respirator-supply-strategies.html						●
d. Train/update medical personnel in use of PPE (including fit testing of N95 or other appropriate respirator)						●

9. Capability to screen staff for illness

	EM National	Federal Gov.	State & Local Gov	State & Local PH	ED	Hosp.
**Action:**						
a. Develop protocol for staff screening including criteria for dismissal from work when symptomatic.						●
b. Consider furlough or reassignment of staff at high-risk for COVID-19 complications						●
c. Develop criteria and process for return to work						●

10. Enhanced facility security and crowd management

	EM National	Federal Gov.	State & Local Gov	State & Local PH	ED	Hosp.
**Action:**						
a. Develop plan and criteria for implementation for enhanced facility security and crowd management including facility lockdown					●	●
b. Establish infected (or potentially infected0 patient flow paths through the institution to avoid contact with uninfected individuals.					●	●
c. Ensure signage at all entry access points with instructions for appropriate triage/treatment areas based on symptoms. Provide appropriate PPE.					●	●
d. Develop plan and criteria for implementation of visitor limitation					●	●
e. Establish a memorandum of understanding (MOU) with law enforcement or other sources for increased institutional security						●

11. Administrative and legal support

	EM National	Federal Gov.	State & Local Gov	State & Local PH	ED	Hosp.
**Action:**						
a. Review and incorporate, as appropriate, guidelines from the American Health Lawyers Association https://www.healthlawyers.org/hlresources/PI/Documents/PI14PFP-eP.pdf#search=flu%20guidelines%20checklist				●	●	●
b. Incorporate provisions of anticipated or actual federal declarations of public health emergency into regional and hospital emergency operations: e.g. possible time-limited waiver of EMTALA, emergency use authorizations (EUAs) for pharmaceuticals			●	●	●	●
c. Establish protocols for rapid credentialing and pre-event credentialing of surge resource personnel						●
d. Incorporate provisions of anticipated or actual state declarations of public health emergency into regional and hospital emergency operations: e.g. possible suspension of destination and diversion policies for EMS providers					●	●
e. Establish legal protocols for human resources to manage attendance of designated mission-critical personnel						●
f. Work with labor and/or staff representatives to develop policies for maintaining staffing, morale, discipline, and safety during emergency operations						●
g. Discuss with legal and medical staff the implementation of "scarce resources allocation" procedures						●
h. Establishment and maintenance of Finance/Administration branch of HICS with assigned functions according to standard protocols and hospital EOPs FEMA rules for reimbursement during an outbreak pandemic – hospital finances http://www.hhs.gov/disasters/discussion/planners/playbook/panflu/subtask.html						●
i. Review legislative authorities of relevance for panflu (e.g. HIPAA, EMTALA, Expanded Scope of Practice, Allocation of Scare Resources. Medical Liability)	●		●		●	●

12. Antiviral prophylaxis and vaccine availability for staff if and when specified by the CDC.

	EM National	Federal Gov.	State & Local Gov	State & Local PH	ED	Hosp.
**Action:**						
a. Perform resource requirement assessment based on the size of your staff and CDC recommendations					●	●
b. Considering federal and state guidance, develop a plan for prioritization and administration of antiviral prophylaxis and vaccine to your staff					●	●
c. Do a resource assessment of what is available in hospital pharmacy, local retail pharmacies and state stockpiles.			●	●		●
d. Develop plans for addressing the gap between estimated requirement and what is expected to be available, including agreements with supply sources			●	●		●

13. Interoperable communications system (fire, law enforcement, EMS, emergency management, receiving hospitals, local/regional public health, local EOC)

	EM National	Federal Gov.	State & Local Gov	State & Local PH	ED	Hosp.
**Action:**						
a. Ensure effective two-way communication among these agencies during an emergency.			●	●	●	●
b. Ensure that dash boards and communications are networked, tested, organized under Incident Command hierarchy, and overseen by a designated regional authority			●	●	●	●

14. Maintaining EMS operations during an outbreak

Action:	EM National	Federal Gov.	State & Local Gov	State & Local PH	ED	Hosp.
a. Review and incorporate, as appropriate, plans and protocols in guidelines for EMS in COVID-19<br>https://www.ems.gov/<br>https://icsw.nhtsa.gov/people/injury/ems/PandemicInfluenzaGuidelines<br>https://files.asprtracie.hhs.gov/documents/aspr-tracie-transport-playbook-508.pdf			●		●	
b. Develop plan to provide for augmentation of staff during an event using additional personnel (volunteer and paid)			●		●	
c. Develop plan for PPE (EMS personnel and patients) acquisition and training			●		●	
d. When recommended by the CDC follow federal and state guidance, develop a plan for prioritization and administration of antiviral prophylaxis to EMS staff and their families			●	●		
e. Establish protocols for alternate patient transport and destinations for non-emergent patients			●	●	●	●
f. Develop protocols for pre-transport patient screening Healthcare Professional Preparedness Checklist For Transport and Arrival of Patients With Confirmed or Possible COVID-19<br>https://www.cdc.gov/coronavirus/2019-ncov/downloads/hcp-preparedness-checklist.pdf	●	●	●			
g. Develop policies and procedures for rapid ambulance decontamination	●	●	●			

15. Laboratory testing protocols

Action:	EM National	Federal Gov.	State & Local Gov	State & Local PH	ED	Hosp.
a. Disseminate guidance to practitioners based on CDC and state health department recommendations and put a system in place to update this information as new data become available.		●	●	●		●
b. Work with your clinical laboratory and public health officials to determine indications for and availability of COVID-19 test kits<br>CDC Tests for COVID-19<br>https://www.cdc.gov/coronavirus/2019-ncov/about/testing.html				●	●	●
c. Work with hospital leadership to develop appropriate inpatient admission testing and patient placement protocols					●	●

16. Alternate locations and staffing for triage and medical screening exams (e.g. mobile units, tents, off-site facility, telemedicine etc.)

Action:	EM National	Federal Gov.	State & Local Gov	State & Local PH	ED	Hosp.
a. Select one or more alternate locations for triage and/or medical screening exams during a pandemic event.			●	●	●	●
b. Develop a staffing plan for the alternate location(s)			●	●	●	●
c. Develop criteria for initiation of use of the alternate location(s)				●	●	●
d. Ensure that any alternate site is designated by the hospital as the appropriate site for a medical screening exam						●
e. Consult Federal Alternate Site guidance https://asprtracie.hhs.gov/technical-resources/48/alternate-care-sites-including-shelter-medical-care/47		●			●	●

17. Off-Site vaccine administration when recommended by the CDC.

Action:	EM National	Federal Gov.	State & Local Gov	State & Local PH	ED	Hosp.
a. Work with public health and emergency management to ensure adequate sites and staffing for vaccine administration to the public			●	●		●

18. Health information call centers

Action:	EM National	Federal Gov.	State & Local Gov	State & Local PH	ED	Hosp.
a. Develop enhanced protocols for COVID-19 telephone triage.				●	●	●
b. Develop protocols for call screening and management at public service answering points (PSAP)				●	●	●
c. Work with local public health to establish social media and telephone center resources to dispense accurate and timely information regarding COVID-19 treatment, home care, criteria for emergency care. https://archive.ahrq.gov/prep/callcenters/				●		●
d. Utilize existing resources such as poison centers or nurse help lines for this purpose				●		●

19. Configuration of ED waiting rooms for distancing

	EM National	Federal Gov.	State & Local Gov	State & Local PH	ED	Hosp.
**Action:**						
a. Configure waiting areas to separate patients with respiratory symptoms from other patients. Ensure signage at all entry access points with instructions for appropriate triage/treatment areas based on symptoms. Provide appropriate PPE.					●	●
b. Maximize distance between individuals with respiratory symptoms up to six feet					●	●
c. Establish protocols for those accompanying patients in the waiting area					●	●

20. Protocols for those visiting patients with fever and respiratory symptoms

	EM National	Federal Gov.	State & Local Gov	State & Local PH	ED	Hosp.
**Action:**						
a. Establish protocols for visitors in treatment areas and inpatient areas					●	●

21. Environmental decontamination capability

	EM National	Federal Gov.	State & Local Gov	State & Local PH	ED	Hosp.
**Action:**						
a. Establish policies and procedures for rapid decontamination of patient treatment areas (including radiology, if necessary)		●	●	●		●

22. Off-site mass screening capability

	EM National	Federal Gov.	State & Local Gov	State & Local PH	ED	Hosp.
**Action:**						
a. Establish local health department sponsorship and staffing plans for mass screening sites				●		
b. Use CDC sanctioned criteria for the self-assessment protocol<br>If you are sick with COVID-19 or suspect you are infected with the virus that causes COVID-19, follow the steps below to help prevent the disease from spreading to people in your home and community. https://www.cdc.gov/coronavirus/2019-ncov/downloads/sick-with-2019-nCoV-fact-sheet.pdf		●		●		

23. Adequate inpatient surge capacity

	EM National	Federal Gov.	State & Local Gov	State & Local PH	ED	Hosp.
**Action:**						
a. Devise protocols for use of alternate care facilities to decompress inpatient units https://asprtracie.hhs.gov/technical-resources/48/alternate-care-sites-including-shelter-medical-care/47						●
b. Establish requirements and investigate process to revise patient staffing ratios						●
c. Identify physical space requirements and capacity (opening unused areas, patient cohorting, doubling up inpatient rooms, canceling elective admits and procedures, and using areas such as post-op recovery for extra critical care space.) Ensure accurate patient tracking.						●
d. Develop alternate staffing and training protocols for ventilator management<br>Project XTREME https://archive.org/details/gov.hhs.ahrq.07-0017-mw.1						●

24. Trained and credentialed volunteers

	EM National	Federal Gov.	State & Local Gov	State & Local PH	ED	Hosp.
**Action:**						
a. Assess the requirements (type and quantity) of medical and non-medical volunteers needed during an outbreak				●	●	●
b. Develop a process for rapid credentialing and just in time training for provider volunteers						●

25. Awareness of SNS surge supplies and capability to receive those supplies

	EM National	Federal Gov.	State & Local Gov	State & Local PH	ED	Hosp.
**Action:**						
a. Work with state and local public health to establish type and quantity of supplies and resources available to mitigate shortages during a pandemic, for example:<br>• Ventilators (including unique operational characteristics)<br>• Antivirals (If/when recommended by the CDC)<br>• PPE<br>• Antibiotic for secondary infection<br>• Vaccine (if/when available)<br>• COVID-19 test kits			●	●	●	●
b. Establish agreements with suppliers to ensure availability (MOU)						●

26. Accurate and coordinated public information dissemination

	EM National	Federal Gov.	State & Local Gov	State & Local PH	ED	Hosp.
**Action:**						
a. Establish protocols for the use of your local/regional/state joint information center that utilizes coordinated health information dispensed by public health officials.			●	●		●
b. Ensure that all personnel adhere to the information dissemination protocols			●			●

27. Augmented post-mortem management and mortuary services

	EM National	Federal Gov.	State & Local Gov	State & Local PH	ED	Hosp.
**Action:**						
a. Establish plans and protocols to augment hospital morgue capacity						●
b. Work with local emergency management and local mortuary services providers to expedite handling of victims			●			●
c. Interim Guidance for Collection and Submission of Postmortem Specimens from Deceased Persons Under Investigation (PUI) for COVID-19, February 2020 https://www.cdc.gov/coronavirus/2019-ncov/hcp/guidance-postmortem-specimens.html			●	●		●

COVID-19 Hyperlinks • Resource List

CDC Guidance – for clinicians on care of patients with COVID-2019
https://www.cdc.gov/coronavirus/2019-ncov/hcp/index.html

Evaluating and Reporting Persons Under Investigation (PUI)
https://www.cdc.gov/coronavirus/2019-nCoV/hcp/clinical-criteria.html

Infection control – COVID-2019
https://www.cdc.gov/coronavirus/2019-ncov/infection-control/index.html

Information on COVID-19 and Pregnant Women and Children
https://www.cdc.gov/coronavirus/2019-ncov/specific-groups/pregnant-women.html

Find your state and local health department
https://www.naccho.org/membership/lhd-directory

COVID-19 Outbreak Tracker. Johns Hopkins
https://gisanddata.maps.arcgis.com/apps/opsdashboard/index.html#/bda7594740fd40299423467b48e9ecf6

World Health Organization (WHO)
https://www.who.int/emergencies/diseases/novel-coronavirus-2019

Interim Guidance for Emergency Medical Services (EMS) Systems and 911 Public Safety Answering Points (PSAPs) for COVID-19 in the United States
https://www.cdc.gov/coronavirus/2019-ncov/hcp/guidance-for-ems.html

Public Readiness and Emergency Preparedness Act
https://www.phe.gov/Preparedness/legal/prepact/Pages/default.aspx

DHHS TRACIE. Coronavirus Topic Collection
https://asprtracie.hhs.gov/technical-resources/44/SARS-MERS/44

COVID-19 Hyperlinks • Resource List

US DHHS National Institutes of Health (NIH) National Library of Medicine (NLM) COVID-19 Resource Page

https://www.nlm.nih.gov/index.html#Novel_Coronavirus

WHO on-line course

https://openwho.org/courses/introduction-to-ncov

Network for Public Health Law. Coronavirus Primer. Authorities, etc

https://www.networkforphl.org/resources/emergency-legal-preparedness-wuhan-coronavirus/

Massachusetts General Hospital. 2019 Novel Coronavirus Toolkit

https://www.massgeneral.org/assets/MGH/pdf/disaster-medicine/SARS-CoV-2%20(COVID-19)%20Toolkit%20 Version%203.pdf

American College of
Emergency Physicians®
ADVANCING EMERGENCY CARE

For more information acep.org/covid19

2008-0320

CDC Archive

This page is archived for historical purposes and is no longer being updated.

This is archived content from the CDC website. The information here may be outdated and links may no longer function. Go to CDC Home for all other recent information.

View in CDC Archive

Go to CDC Archive Home Page

COVID-19 Pandemic Planning Scenarios

Updated Mar. 19, 2021

Summary of Recent Changes

Updates as of March 19, 2021:

- The Infection Fatality Ratio (IFR) parameter has been updated to reflect recently published estimates. This parameter is now presented as the number of deaths per 1,000,000 infections for ease of interpretation.
- The healthcare utilization statistics in Table 2 have been updated to include a 0–17-years-old age group.
- This will be the final update to the COVID-19 Pandemic Planning Scenarios, as there is now a substantial body of published literature that modelers can draw on to inform parameter estimates and assumptions for their models for the general population and for sub-populations of interest. In addition, CDC has several sources that will continue to update COVID-19-related data over time, including COVID Data Tracker, COVID-19 Case Surveillance Public Use Data, and COVID-19-Associated Hospitalization Surveillance Network (COVID-NET).

CDC and the Office of the Assistant Secretary for Preparedness and Response (**ASPR**) have developed five COVID-19 Pandemic Planning Scenarios that are designed to advance public health preparedness and planning and help inform decisions by public health officials who use mathematical modeling and by mathematical modelers throughout the federal government. Models developed using the data provided in the Planning Scenario tables can help evaluate the potential effects of different community mitigation strategies (e.g., social distancing). The Planning Scenarios may also be useful to hospital administrators in assessing resource needs and can be used in conjunction with the COVID-19 Surge Tool.

Each Planning Scenario is based on a set of numerical values for the biological and epidemiological characteristics of COVID-19 illness, which is caused by the SARS-CoV-2 virus. These values—called *parameter values*—can be used in models to estimate the possible effects of COVID-19 in U.S. states and localities. This document was first posted on May 20, 2020, with the understanding that the parameter values in each Scenario would be updated and augmented over time as we learn more about the epidemiology of COVID-19. This will be the final update of the COVID-19 Pandemic Planning Scenarios, as there is now a substantial body of published literature that modelers can draw on to inform parameter estimates and assumptions for their models. In addition, CDC has several sources that will continue to update COVID-19-related data over time, including:

- COVID Data Tracker is the repository for CDC's COVID-19 data. COVID Data Tracker combines data from across the response and provides summary statistics by category (e.g., cases and deaths, testing, and vaccinations). These data are updated daily.

- COVID-19 Case Surveillance Public Use Data are deidentified line-level data from COVID-19 cases reported to CDC. This includes data on demographics and clinical information (e.g., symptom-onset date and hospital status) and is updated monthly. In addition, a restricted-use version of these data, which includes county and state information, is available to users who complete a registration process, sign a data use agreement, and obtain approval from CDC.
- COVID-NET is a population-based surveillance system that collects data on laboratory-confirmed COVID-19-associated hospitalizations through a network of more than 250 acute care hospitals in 14 states. COVID-NET provides information on age-specific clinical outcomes as well as age- and location-specific COVID-19 hospitalization rates and are updated weekly.

In this final update, the age-specific estimates of Infection Fatality Ratios (IFRs) have been updated to reflect recently published estimates of IFRs from a systematic review and meta-analysis.[1] These updated estimates have a wider uncertainty range to better reflect the potential variation in IFR geographically and over time. These values are intended to capture the national-level burden of COVID-19 deaths; however, national-level estimates may not reflect region-specific IFRs. Therefore, caution should be used when applying suggested IFR values to specific states, counties, and cities. This update also includes parameter values for healthcare utilization in individuals aged 0–17-years-old.

New data on COVID-19 are available daily, yet information about the biological aspects of SARS-CoV-2 and epidemiological characteristics of COVID-19 remain limited, and uncertainty remains around nearly all parameter values. For example, current estimates of IFRs do not account for time-varying changes in hospital capacity (e.g., bed capacity, ventilator capacity, or workforce capacity) or for differences in case ascertainment in congregate and community settings or in rates of underlying health conditions that may contribute to a higher frequency of severe illness in those settings. A nursing home, for example, may have a high incidence of infection (because of close contacts among many individuals) and severe disease (because of a high rate of underlying conditions) that does not reflect the frequency or severity of disease in the broader population of older adults. In addition, the practices for testing nursing home residents for SARS-CoV-2 upon identification of a positive resident may be different than testing practices for contacts of confirmed cases in the community. Observed parameter values may also change over time. For example, the percentage of transmission occurring before symptom onset will be influenced by how quickly and effectively both symptomatic people and the contacts of known individuals with COVID-19 (cases) are quarantined. In addition, observed parameter values may be influenced by the recent emergence of novel SARS-CoV-2 variants.

The parameters in the Planning Scenarios:

- Are estimates intended to support public health preparedness and planning;
- Are **not** predictions of the expected effects of COVID-19;
- Do **not** reflect the impact of any behavioral changes, social distancing, or other interventions; and
- Do **not** reflect the impact of the emergence of novel SARS-CoV-2 variants.

The Five Scenarios

The five COVID-19 Pandemic Planning Scenarios (Box 1) represent a range of possible parameters for COVID-19 in the United States. All parameter values are based on current COVID-19 surveillance data and scientific knowledge.

- Scenarios 1 through 4 are based on parameter values that represent the lower and upper bounds of disease severity and viral transmissibility. The parameter values used in these Pandemic Planning Scenarios are likely to change as we obtain additional data about the upper and lower bounds of disease severity and the transmissibility of SARS-CoV-2, the virus that causes COVID-19.
- Scenario 5 represents a current best estimate about viral transmission and disease severity in the United States, with the same caveat: the parameter values will change as more data become available.

Parameter values that vary among the Pandemic Planning Scenarios are listed in Table 1, while parameter values common to all five scenarios are listed in Table 2. Definitions of the parameters are provided below, and the source for each parameter value is indicated in the Tables.

The Parameter Values: Definitions

Parameter values that vary across the five COVID-19 Pandemic Planning Scenarios (Table 1) include measures of viral transmissibility, disease severity, and pre-symptomatic and asymptomatic disease transmission. Age-stratified estimates are provided, where sufficient data are available.

Viral Transmissibility

- **Basic reproduction number (R_0):** The average number of people that one person with SARS-CoV-2 is likely to infect in a population without any immunity (from previous infection) or any interventions. R_0 is an estimate of the average transmissibility in a completely naïve population. R_0 estimates vary across populations and are a function of the duration of contagiousness, the likelihood of infection per contact between a susceptible person and an infectious person, and the contact rate.[2,3] A separate but related parameter is the effective or time-varying reproduction number (R_e or R_t), which estimates the average transmission in a population with mitigation measures and immunity.

Disease Severity

- **Infection Fatality Ratio (IFR):** The number of individuals who **die** of the disease among all infected individuals (symptomatic and asymptomatic). This parameter is not necessarily equivalent to the number of reported deaths per reported case because many cases and deaths are never confirmed to be COVID-19 and there is a lag in time between when people are infected and when they die. This parameter also reflects the existing standard of care, which might vary by location or hospital and could be affected by the introduction of new therapeutics. The IFR values presented in Table 1 are intended to capture the national-level burden of COVID-19 deaths; however, these values may not reflect IFR in specific states, counties, or cities in the United States.

Presymptomatic and Asymptomatic Contribution to Disease Transmission

A **presymptomatic case** of COVID-19 is an individual infected with SARS-CoV-2 who has not yet exhibited symptoms at the time of testing but who later exhibits symptoms during the course of the infection. An **asymptomatic case** is an individual infected with SARS-CoV-2 who does not exhibit symptoms at any time during the course of infection. Parameter values that measure the presymptomatic and asymptomatic contribution to disease transmission include:

- **Percentage of infections that are asymptomatic:** The percentage of persons who are infected with SARS-CoV-2 but never show symptoms of the disease. Asymptomatic cases are challenging to identify because individuals do not know they are infected unless they are tested over the course of their infection, which is typically done systematically only as a part of a scientific study.
- **Infectiousness of asymptomatic individuals relative to symptomatic individuals:** The contribution to transmission of SARS-CoV-2 from asymptomatic individuals compared to the contribution to transmission of SARS-CoV-2 from symptomatic individuals. For example, a parameter value of 50% means that an asymptomatic individual is half as infectious as a symptomatic individual, whereas a parameter value of 100% means that an asymptomatic individual is just as likely to transmit infection as a symptomatic individual.
- **Percentage of transmission occurring before symptom onset:** Among symptomatic cases, the percentage of new cases of COVID-19 due to transmission from a person with COVID-19 who infects others before exhibiting symptoms (presymptomatic).

Parameter values that do not vary across the five Pandemic Planning Scenarios (Table 2) are:

- **Level of pre-existing immunity to COVID-19 in the community:** The percentage of the U.S. population with existing immunity to COVID-19 before the start of the pandemic, which began in late 2019.
- **Ratio of estimated infections to reported case counts:** The estimated number of infections divided by the number of reported cases. The level of case detection likely varies by the age distribution of cases, location, and over time.
- **Time from exposure to symptom onset:** The number of days from the time a person has contact with an infected person that results in COVID-19 infection and the first appearance of symptoms.
- **Time from symptom onset in an individual and symptom onset of a second person infected by that individual:** The number of days from the time a person becomes symptomatic and when the person who they infect becomes symptomatic.

Additional parameter values common to the five COVID-19 Pandemic Planning Scenarios are these 10 measures of healthcare usage:

- Median number of days from symptom onset to SARS-CoV-2 test among SARS-CoV-2–positive patients

- Median number of days from symptom onset to SARS-CoV-2 test among SARS-CoV-2–positive patients
- Median number of days from symptom onset to hospitalization
- Median number of days of hospitalization among those not admitted to the intensive care unit (ICU)
- Median number of days of hospitalization among those admitted to the ICU
- Percentage of patients admitted to the ICU among those hospitalized
- Percentage of patients on mechanical ventilation among those hospitalized (includes both non-ICU and ICU admissions)
- Percentage of patients who die among those hospitalized (includes both non-ICU and ICU admissions)
- Median number of days on mechanical ventilation
- Median number of days from symptom onset to death (for patients who die)
- Median number of days from death to reporting of that death

These healthcare-related parameters (Table 2) assist in the assessment of resource needs as the pandemic progresses.

Box 1 Description of the Five COVID-19 Pandemic Planning Scenarios

For each Pandemic Planning Scenario:

- Parameter value for **viral transmissibility** is the Basic Reproduction Number (R_0)
- Parameter value for **disease severity** is the Infection Fatality Ratio (IFR)
- Parameter values for the **presymptomatic and asymptomatic contribution** to disease transmission are:
  - Percentage of transmission occurring before the symptom onset (from presymptomatic individuals)
  - Percentage of infections that are asymptomatic
  - Infectiousness of asymptomatic individuals relative to symptomatic individuals

For Pandemic Scenarios 1-4:

- These Scenarios are based on parameter values that represent the lower and upper bounds of disease severity and viral transmissibility. The parameter values used in these Scenarios are likely to change as we obtain additional data about the upper and lower bounds of disease severity and viral transmissibility of COVID-19.

For Pandemic Scenario 5:

- This Scenario represents a current best estimate about viral transmission and disease severity in the United States, with the same caveat: The parameter values will change as more data become available.

Scenario 1:

- Lower-bound values for virus transmissibility and disease severity
- Lower percentage of transmission before the onset of symptoms
- Lower percentage of infections that never have symptoms and lower contribution of those cases to transmission

Scenario 2:

- Lower-bound values for virus transmissibility and disease severity
- Higher percentage of transmission before the onset of symptoms
- Higher percentage of infections that never have symptoms and higher contribution of those cases to transmission

Scenario 3:

- Upper-bound values for virus transmissibility and disease severity
- Lower percentage of transmission before the onset of symptoms
- Lower percentage of infections that never have symptoms and lower contribution of those cases to transmission

Scenario 4:

Scenario 4:

- Upper-bound values for virus transmissibility and disease severity
- Higher percentage of transmission before the onset of symptoms
- Higher percentage of infections that never have symptoms and higher contribution of those cases to transmission

Scenario 5:

- Parameter values for disease severity, viral transmissibility, and presymptomatic and asymptomatic disease transmission that represent the best estimate, based on the latest surveillance data and scientific knowledge.

Table 1. Parameter Values that vary among the five COVID-19 Pandemic Planning Scenarios. The scenarios are intended to advance public health preparedness and planning. They are **not** predictions or estimates of the expected impact of COVID-19.

Parameter	Scenario 1	Scenario 2	Scenario 3	Scenario 4	Scenario 5: Current Best Estimate
**R_0***	2.0		4.0		2.5
**Infection fatality ratio (Estimated number of deaths per 1,000,000 infections)†**	0–17 years old: 6<br>18–49 years old: 150<br>50–64 years old: 1,800<br>65+ years old: 26,000		0–17 years old: 80<br>18–49 years old: 1,700<br>50–64 years old: 20,000<br>65+ years old: 270,000		0–17 years old: 20<br>18–49 years old: 500<br>50–64 years old: 6,000<br>65+ years old: 90,000
**Percent of infections that are asymptomatic§**	15%	70%	15%	70%	30%
**Infectiousness of asymptomatic individuals relative to symptomatic^**	25%	100%	25%	100%	75%
**Percentage of transmission occurring prior to symptom onset****	30%	70%	30%	70%	50%

* The best estimate representative of the point estimates of R0 from the following sources:

- Chinazzi M, Davis JT, Ajelli M, *et al*. The effect of travel restrictions on the spread of the 2019 novel coronavirus (COVID-19) outbreak. *Science*. 2020;368(6489):395–400; Imai N, Cori A, Dorigatti I, *et al*. (2020). Report 3: Transmissibility of 2019-nCoV. *Online report*
- Li Q, Guan X, Wu P, *et al*. Early transmission dynamics in Wuhan, China, of novel coronavirus-infected pneumonia. *N Engl J Med*. 2020;382(13):1199–1207.
- Munayco CV, Tariq A, Rothenberg R, *et al*. Early transmission dynamics of COVID-19 in a southern hemisphere setting: Lima-Peru: February 29th-March 30th, 2020. *Infect Dis Model*. 2020;5:338–345.
- Salje H, Tran Kiem C, Lefrancq N, *et al*. Estimating the burden of SARS-CoV-2 in France *Science* 2020;81(5):816-846.

The range of estimates for Scenarios 1–4 represent the upper and lower bound of the widest confidence interval estimates reported in: Li Q, Guan X, Wu P, *et al*. Early transmission dynamics in Wuhan, China, of novel coronavirus-infected pneumonia. *N Engl J Med*. 2020;382(13):1199–1207.

Substantial uncertainty remains around the R0 estimate. Notably, Sanche S, Lin YT, Xu C, *et al*. High contagiousness and rapid spread of severe acute respiratory syndrome coronavirus 2. *Emerg Infect Dis*. 2020;26(7):1470–1477. This study estimated a median R0 value of 5.7 in Wuhan, China. In an analysis of eight European countries and the United States, the same group estimated R0 of between 4.0 and 7.1 in the preprint manuscript: Ke R, Sanche S, Romero-Severson E, Hengartner N. (2020). Fast spread of COVID-19 in Europe and the United States suggests the necessity of early, strong, and comprehensive interventions. *medRxiv*.

† These estimates are based on age-specific estimates of infection fatality ratios from Levin AT, Hanage WP, Owusu-Boaitey N, *et al*. Assessing the age specificity of infection fatality rates for COVID-19: Systematic review, meta-analysis, and public policy implications. *Euro J Epidemiol*. 2020;35(12):1123–1135.

Using a meta regression of data from England, France, Ireland, Italy, Netherlands, Portugal, Spain, Geneva (Switzerland), Belgium, Sweden, Ontario (Canada), and 12 U.S. locations (Atlanta, Georgia; Connecticut; Indiana; Louisiana; Miami;, Minneapolis, Minnesota; Missouri; New York; Philadelphia, Pennsylvania; Salt Lake City, Utah; San Francisco, California; and Seattle, Washington), Levin *et al*. produced estimates of IFR and associated 95% confidence intervals for 0.5–year age bands from 1 to 96 years old. To obtain the estimated values for each scenario, the IFR estimates by age were averaged to broader age groups, using weights based on the age distribution of cases from COVID-19 Case Surveillance Data reported by February 14, 2021 (public use version of data: https://data.cdc.gov/Case-Surveillance/COVID-19-Case-Surveillance-Public-Use-Data/vbim-akqf).

§ The percent of cases that are asymptomatic (i.e., never experience symptoms) remains uncertain. Longitudinal testing of individuals is required to accurately detect the absence of symptoms for the full period of infectiousness. Current peer-reviewed and preprint studies vary widely in follow-up times for re-testing, or do not include re-testing of cases. Additionally, studies vary in the definition of a symptomatic case, which makes it difficult to make direct comparisons between estimates. Furthermore, the percent of cases that are asymptomatic may vary by age, and the age groups reported in the studies can vary.

Given these limitations, the range of estimates for Scenarios 1–4 is wide. The lower-bound estimate approximates the lower 95% confidence interval bound estimated from: Byambasuren O, Cardona M, Bell K, Clark J, McLaws ML, Glasziou P. Estimating the extent of asymptomatic COVID-19 and its potential for community transmission: Systematic review and meta-analysis. *Official Journal of the Association of Medical Microbiology and Infectious Disease Canada* 2020;5(4):223–234. The upper-bound estimate approximates the upper 95% confidence interval bound estimated from: Poletti P, Tirani M, Cereda D, *et al*. (2020). Probability of symptoms and critical disease after SARS-CoV-2 infection. *arXiv preprint arXiv:2006.08471*. The best estimate aligns with estimates from:

- Oran DP, Topol EJ. Prevalence of asymptomatic SARS-CoV-2 infection: A narrative review. *Ann Intern Med*. 2020;173(5):362–367.
- Oran DP, Topol EJ. The proportion of SARS-CoV-2 infections that are asymptomatic: A systematic review. [published online ahead of print, 2021 January 22] *Ann Intern Med*.
- Buitrago-Garcia D, Egli-Gany D, Counotte MJ, *et al*. Occurrence and transmission potential of asymptomatic and presymptomatic SARS-CoV-2 infections: A living systematic review and meta-analysis. *PLoS medicine*, 2020;17(9):e1003346.
- Ravindra K, Malik VS, Padhi BK, Goel S, and Gupta M. (2020) Consideration for the asymptomatic transmission of COVID-19: Systematic review and meta-analysis. *medRxiv*.
- Beale S, Hayward A, Shallcross L, Aldridge RW, and Fragaszy E. (2020) A rapid review of the asymptomatic proportion of PCR-confirmed SARS-CoV-2 infections in community settings. *medRxiv*.

^ The current best estimate is based on multiple assumptions. The relative infectiousness of asymptomatic cases to symptomatic cases remains highly uncertain, as asymptomatic cases are difficult to identify and transmission is difficult to observe and quantify. The estimates for relative infectiousness are assumptions based on studies of viral shedding dynamics. The upper bound of this estimate reflects studies that have shown similar durations and amounts of viral shedding between symptomatic and asymptomatic cases:

- Lee S, Kim T, Lee E, *et al*. Clinical course and molecular viral shedding among asymptomatic and symptomatic patients with SARS-CoV-2 infection in a community treatment center in the Republic of Korea. *JAMA Intern Med.* 2020;180(11):1–6.
- Zou L, Ruan F, Huang M, *et al*. SARS-CoV-2 viral load in upper respiratory specimens of infected patients. *N Engl J Med*. 2020;382(12):1177–1179.
- Zhou R, Li F, Chen F, *et al*. Viral dynamics in asymptomatic patients with COVID-19. *Int J Infect Dis*. 2020;96:288–290.

The lower bound of this estimate reflects data indicating that viral loads are higher in severe cases relative to mild cases (Liu Y, Yan LM, Wan L, *et al*. Viral dynamics in mild and severe cases of COVID-19. *Lancet Infect Dis*. 2020;20(6):656–657) and data showing that viral loads and shedding durations are higher among symptomatic cases relative to asymptomatic cases (Noh JY, Yoon JG, Seong H, *et al*. Asymptomatic infection and atypical manifestations of COVID-19: Comparison of viral shedding duration. *J Infect*. 2020;81(5):816–846.

** The lower bound of this parameter is approximated from the lower 95% confidence interval bound from: He X, Lau EH, Wu P, *et al*. Temporal dynamics in viral shedding and transmissibility of COVID-19. *Nature Med.* 2020;26(5):672–675. The upper bound of this parameter is approximated from the higher estimates of individual studies included in: Casey M, Griffin J, McAloon CG, *et al*. (2020). Estimating presymptomatic transmission of COVID-19: A secondary analysis using published data. *medRxiv.* The best estimate is the geometric mean of the point estimates from these two studies and aligns with estimates from:

- Moghadas SM, Fitzpatrick MC, Sah P, *et al*. The implications of silent transmission for the control of COVID-19 outbreaks. *Proc Natl Acad Sci USA*. 2020;117(30):17513–17515.
- Johansson MA, Quandelacy TM, Kada S, *et al*. 2021. SARS-CoV-2 transmission from people without COVID-19 symptoms. *JAMA Network Open* 2021;4(1):e2035057-e2035057.

Table 2. Parameter Values Common to the Five COVID-19 Pandemic Planning Scenarios. The parameter values are likely to change as we obtain additional data about disease severity and viral transmissibility of COVID-19.

Parameter values are based on data received by CDC between December 31, 2020, and February 14, 2021, including COVID-19 Case Surveillance Data (public use version of data: https://data.cdc.gov/Case-Surveillance/COVID-19-Case-Surveillance-Public-Use-Data/vbim-akqf); data from the Hospitalization Surveillance Network (COVID-NET) (through December 31, 2020); and data from Human and Health Services Protect (*HHS Protect*) (through February 14, 2020).

**Pre-existing immunity**<br>**Assumption, ASPR and CDC**	No pre-existing immunity before the pandemic began in 2019. It is assumed that all members of the U.S. population were susceptible to infection prior to the pandemic.
**Time from exposure to symptom onset***	~6 days (mean)
**Time from symptom onset in an individual and symptom onset of a second person infected by that individual†**	~6 days (mean)
**Mean ratio of estimated infections to reported case counts, overall (range)§**	11 (6, 24)
**Parameter Values Related to Healthcare Usage**	
**Median number of days from symptom onset to SARS-CoV-2 test among SARS-CoV-2 positive patients (interquartile range)^**	Overall: 2 (0, 4) days

**Median number of days from symptom onset to hospitalization (interquartile range)****	0–17 years old: 2 (0, 7) days<br>18–49 years old: 6 (2, 10) days<br>50–64 years old: 6 (2, 10) days<br>≥65 years old: 4 (1, 9) days
**Median number of days of hospitalization among those not admitted to ICU (interquartile range) ††**	0–17 years old: 2 (1, 4) days<br>18–49 years old: 3 (2, 6) days<br>50–64 years old: 4 (2, 7) days<br>≥65 years old: 5 (3, 9) days
**Median number of days of hospitalization among those admitted to the ICU (interquartile range)††,§§**	0–17 years old: 5 (2, 10.5) days<br>18–49 years old: 10 (6, 20) days<br>50–64 years old: 14 (8, 25) days<br>≥65 years old: 13 (7, 22) days
**Percent admitted to the ICU among those hospitalized††**	0–17 years old: 27.5%<br>18–49 years old: 18.9%<br>50–64 years old: 27.1%<br>≥65 years old: 26.9%
**Percent on mechanical ventilation among those hospitalized. Includes both non-ICU and ICU admissions††**	0–17 years old: 5.8%<br>18–49 years old: 9.0%<br>50–64 years old: 15.1%<br>≥65 years old: 15.6%
**Percent that die among those hospitalized. Includes both non-ICU and ICU admissions††**	0–17 years old: 0.7%<br>18–49 years old: 2.1%<br>50–64 years old: 7.9%<br>≥65 years old: 18.8%
**Median number of days of mechanical ventilation (interquartile range)****	Overall: 5 (2, 11) days
**Median number of days from symptom onset to death (interquartile range)****	0–17 years old: 10 (4, 31) days<br>18–49 years old: 17 (10, 30) days<br>50–64 years old: 19 (11, 30) days<br>≥65 years old: 16 (9, 25) days
**Median number of days from death to reporting (interquartile range)^^**	0–17 years old: 8 (3, 33) days<br>18–49 years old: 26 (5, 63) days<br>50–64 years old: 28 (5, 64) days<br>≥65 years old: 23 (4, 59) days

* McAloon C, Collins Á, Hunt K, *et al*. Incubation period of COVID-19: A rapid systematic review and meta-analysis of observational research. *BMJ Open*. 2020;10(8):e039652; Ma S, Zhang J, Zeng M, *et al*. Epidemiological parameters of COVID-19: Case series study. *J Med Internet Res.* 2020;22(10):e19994.

† He X, Lau EH, Wu P, *et al*. Temporal dynamics in viral shedding and transmissibility of COVID-19. *Nat Med.* 2020;26(5):672–675; Saurabh S, Verma MK, Gautam V, *et al*. Transmission dynamics of the COVID-19 epidemic at the district level in India: Prospective observational study. *JMIR Public Health Surveill*. 2020;6(4):e22678.

§ The point estimate is the geometric mean of the location-specific point estimates of the ratio of estimated infections to reported cases, from Havers FP, Reed C, Lim T, *et al*. Seroprevalence of antibodies to SARS-CoV-2 in 10 sites in the United States, March 23-May 12, 2020. *JAMA Intern Med*. 2020 Jul 12. doi: 10.1001/jamainternmed.2020.4130. The lower and

upper bounds for this parameter estimate are the lowest and highest point estimates of the ratio of estimated infections to reported cases, respectively.

^ Estimates only include symptom onset dates during March 1, 2020 – January 31, 2021, to ensure cases have had sufficient time to obtain SARS-CoV-2 tests. Estimates represent time to obtain SARS-CoV-2 tests among cases who tested positive for SARS-CoV-2. Estimates are based on line-level case surveillance data reported to CDC.

** Estimates only include symptom onset dates during March 1, 2020 – January 31, 2021, to ensure cases have had sufficient time to observe the outcome (hospital discharge or death).

†† Based on data reported to COVID-NET by December 31, 2020. https://gis.cdc.gov/grasp/COVIDNet/COVID19_5.html

§§ Cumulative length of stay for persons admitted to the ICU, inclusive of both ICU and non-ICU days.

^^ Estimates only include death dates between March 1, 2020 – January 31, 2021, to ensure sufficient time for reporting.

References

1. Levin AT, Hanage WP, Owusu-Boaitey N, *et al.* Assessing the age specificity of infection fatality rates for COVID-19: Systematic review, meta-analysis, and public policy implications. *Euro J Epidemiol*. 2020;35(12):1123–1138.
2. Dietz K. The estimation of the basic reproduction number for infectious diseases. *Stat Methods Med Res*. 1993;2:23–41.
3. Delamater PL, Street EJ, Leslie TF, *et al*. Complexity of the basic reproduction number (R0). *Emerg Infect Dis*. 2019;25(1):1

Archive

COVID-19 Pandemic Planning Scenarios – May 20, 2020 [7 pages]

COVID-19 Pandemic Planning Scenarios – July 10, 2020 [9 pages]

COVID-19 Pandemic Planning Scenarios – September 10, 2020 [9 Pages]

Last Updated Mar. 19, 2021
