# Supplementary material for: Comparative analysis of emergency department admissions: A multi-center study on patient characteristics and mortality before and during the early phase of pandemic in Turkey
Source: Medicine (Baltimore). 2025 Sep 12;104(37):e44438. doi: 10.1097/MD.0000000000044438 (PMC12440398; doi:10.1097/MD.0000000000044438)

Figure S1. Daily Confirmed COVID-19 Cases per Million People (*present study period)


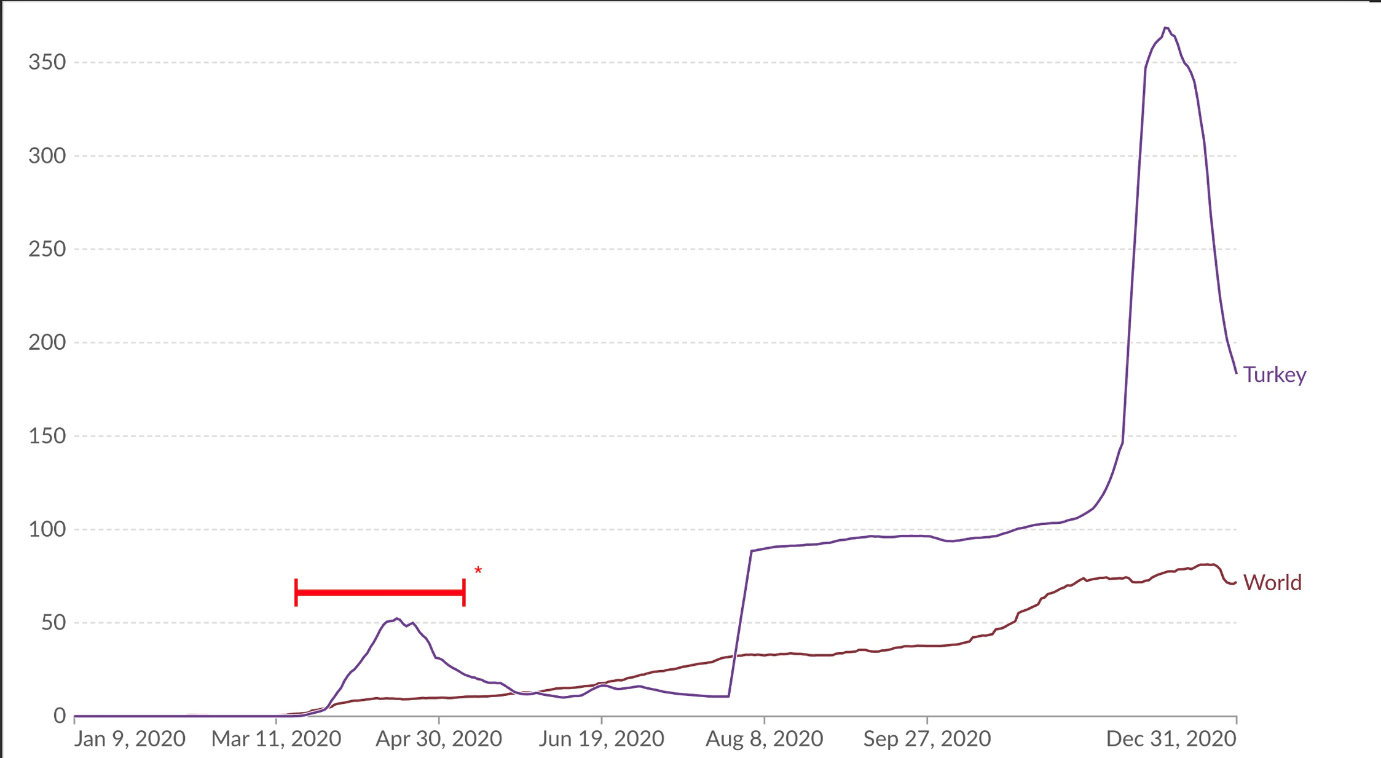


Figure S2. Daily ED Admission Numbers During and Pre-Pandemic Periods


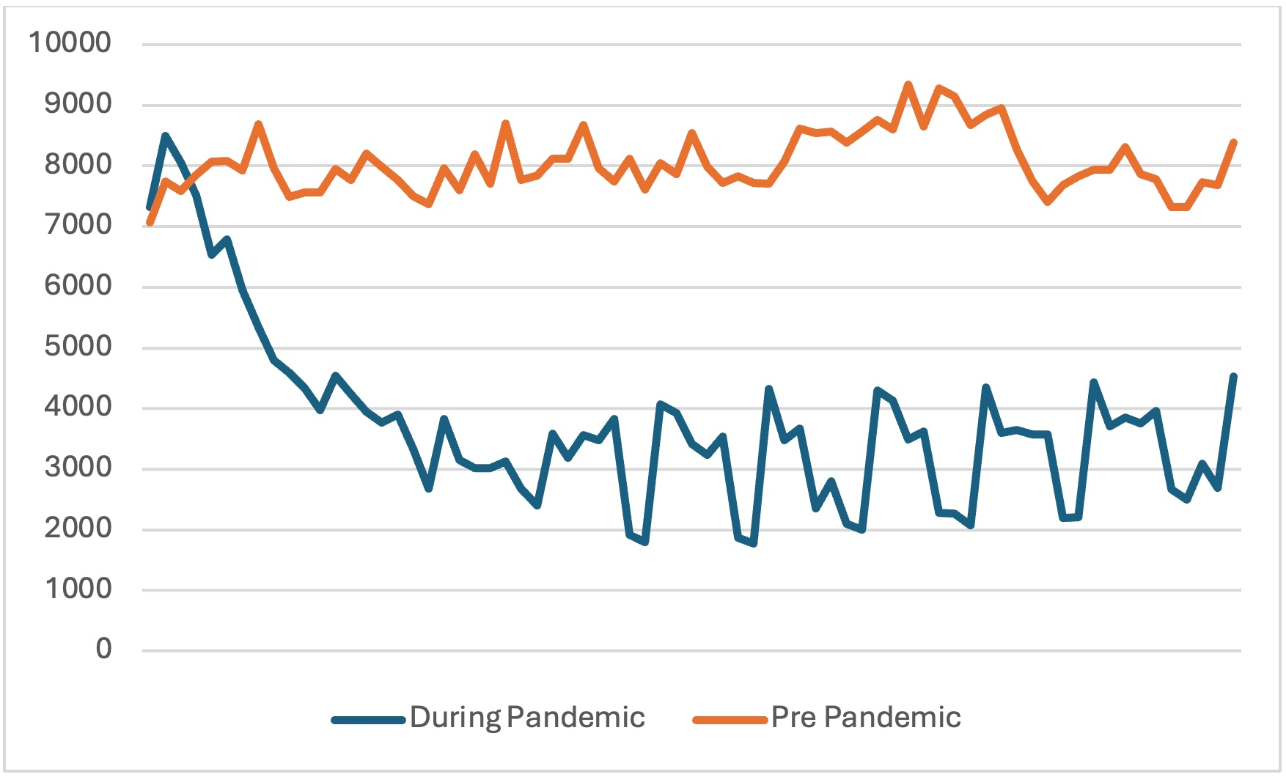

Supplement: Supplementary file 2 [file medi-104-e44438-s002.docx]
